# Patient discourses on real-time access to test results via hospital portals: a discourse analysis of semistructured interviews with Dutch patients

**DOI:** 10.1136/bmjopen-2024-088201

**Published:** 2024-11-24

**Authors:** Pauline Hulter, Anne Marie J W M Weggelaar-Jansen, Kees Ahaus, Bettine Pluut

**Affiliations:** 1 Erasmus School of Health Policy & Management, Department Health Services Management & Organization, Erasmus University Rotterdam, Rotterdam, The Netherlands; 2 Tranzo, Scientific Center for Care and Wellbeing, Tilburg School of Social and Behavioral Sciences, Tilburg University, Tilburg, The Netherlands; 3 Clinical Informatics, Eindhoven University of Technology, Eindhoven, The Netherlands

**Keywords:** eHealth, Patient-Centered Care, Patients

## Abstract

**Abstract:**

**Objective:**

Real-time access to test results on patient portals can have advantages and disadvantages for patients. It confronts patients with a complicated decision, namely whether to consult results before the medical consultation. To gain a deep understanding of patients’ decision-making processes, we unravelled three discourses about real-time access to test results, each of which articulates a different set of values, assumptions and arguments. Our research question was what patient discourses on real-time access to test results can be distinguished?

**Design:**

We conducted discourse analysis on 28 semistructured interviews.

**Setting:**

Interviews were conducted with patients who had (no) experience with real-time access to test results. Our participants were treated in different hospitals, and therefore, used different portals since Dutch hospitals can choose from suppliers for their patient portals.

**Participants:**

Patients with experience (n=15) and without experience (n=13) of real-time access to test results on a patient portal.

**Results:**

We identified three discourses: (1) real-time access as a source of stress, which highlighted how real-time access could cause stress due to the complexity of deciding whether to access test results, the incomprehensibility of medical language and the urge to repeatedly check if test results were available, (2) anxiety reduction through real-time access showed how real-time access can reduce stress by reducing waiting times and (3) real-time access for self-management showed how real-time access can give patients an opportunity for self-management because they can make informed decisions and are better prepared for the medical consultation.

**Conclusion:**

Our study shows the plurality in opinions on real-time access, which helps in forming different strategies to inform and support patients in order to realise optimal use of real-time access.

**STRENGTHS AND LIMITATIONS OF THIS STUDY**

- We conducted semistructured interviews over time with 28 participants who provided a comprehensive overview of opinions and experiences on real-time access to test results.
- Three researchers conducted a thorough theory-informed coding process that led to a deeper understanding of the phenomenon under study.
- Our sample might be biased since we used the snowball method for recruitment.
- However, the sample is well-balanced since we interviewed patients both without (n=13) and with (n=15) experience with real-time access to test results.
- Participants often referred to ‘other people’ instead of sharing their own views/experiences which might potentially affect the validity of our findings.

## Introduction

 Patient portals can be defined as ‘*provider-tethered applications that allow patients to access, but not control, certain health care information (eg, their electronic health record) and provide communication and administrative functions […]’* (p.2).^1^ Accordingly, a patient portal offers three services to patients: (1) health-related information from one provider (eg, a healthcare organisation), (2) organisational information of this particular provider and (3) communication with the healthcare professionals of this provider.^2^,^5^

One of the most popular patient portal services is providing health-related information such as test results.^6^,^9^ National digital health patient portals vary by country, as do the requirements for disclosing test results.^10^ In the Netherlands, healthcare providers can decide when patients can access test results on the portal. A recent scoping review shows this is still an important topic.^11^ Providers can choose to disclose results in real time (immediate release) or after a delay (up to 28 days).^12^,^17^ This decision presents challenges for patients and professionals. If the provider chooses to disclose test results in real time, the patients can see their results before consulting the doctor, which might cause negative experiences like anxiety and incomprehensibility^14 15 18 19^ or positive experiences like reassurance,^18^ a sense of ownership over their results^7^ and better preparation for the medical consultation.^1 14 18 20^

Multiple scholars have studied the advantages and disadvantages of real-time access to test results on a patient portal for patients and healthcare professionals. The literature identified three advantages. First, patients are better able to process their test results at home and prepare for the outpatient consultations with their healthcare professional,^1820^,^22^ for example, by preparing questions.^14 21^ Second, patients develop a strong sense of ownership in relation to their results because they can decide for themselves when to access them^12^ and become more informed prior to the medical consultation.^12 14 23^ This enhances shared decision-making with their healthcare professional.^13^ Third, patients can review their results and contact their healthcare professional earlier if their results are abnormal, which enhances patient safety.^12^ A disadvantage could be that patients may find it difficult to interpret their test results^12^,^1424^ potentially leading to (unnecessary) anxiety,^12 14 15 23 25^ stress^22^ and confusion.^15^

These advantages and disadvantages signal that deciding when to disclose test results is not a simple technological choice made by the healthcare provider and/or the patient. Instead, the possible advantages and disadvantages, which influence patient involvement, patient safety, patient empowerment, patient-centred care and patient satisfaction, must be considered. Literature has shown that looking at test results before an outpatient consult allows patients to better prepare questions for their consultation.^14 18 21^ At the same time, real-time access could cause anxiety if the information shared is misunderstood.^15^ Therefore, the timing of test result disclosure is ambiguous and needs careful consideration.^26^ A thorough exploration of how patients perceive real-time access and make decisions is both interesting and highly relevant.

In a systematic review on engaging patients in their own care process using eHealth, researchers concluded that a profound understanding of patients’ experiences with eHealth technologies is often not achieved, despite its importance.^27^ Similar difficulties have been experienced with research into real-time access to test results. Studies have used questionnaires,^15 20^ literature reviews,^12^ discussion papers,^12 23^ quantitative data from a patient portal,^28^ reported incidents by healthcare professionals, patients’ complaints at the complaint commission and portal helpdesk^15^ or mixed methods^11^ to investigate this topic. Still, little is known about the expectations, experiences and emotions of patients in relation to their norms and values.^11^ Consequently, we have performed a qualitative study of how patients construct real-time access to test results.

A discourse analysis of scientific publications on patient-centredness signals how patient norms, values and constructions of ‘good care’ influence their attitude toward eHealth technologies.^29^ Consequently, we decided to perform a discourse analysis of the in-depth interviews we conducted with patients on their feelings, thoughts, expectations and experiences of real-time access to test results in relation to their view of patient-centred care. To the best of our knowledge, a discourse analysis of interviews with patients on real-time access to test results on a patient portal has not been conducted. A discourse can be defined as ‘*an interrelated set of texts and practices that bring an object into being’* (p. 3, Parker, 1992).^30^ This microlevel discourse analysis gave us a deeper understanding of the feelings, thoughts, expectations and experiences of real-time access to test results from a patient perspective. The benefit of a discourse analysis is that it helps us understand patients’ perspectives on real-time access by studying them in the broader context of their lived experiences and their views of what patient-centred care entails.^31^

Discourse analysis can reveal the differences in patients’ experiences, thereby revealing how reality is produced within the patient context, and how this reality influences the patient’s understanding and actions.^30 32^ In addition, it helps to investigate the practical consequences of different discourses on real-time patient access^33 34^ in two ways. First, it highlights the different policy options and their implications during policy development for healthcare providers. Second, it shows how the design of technologies, healthcare processes and information materials can help patients and healthcare professionals to make decisions about real-time access. Besides investigating practical consequences, the aim of our study was to obtain a deeper understanding of patients’ feelings, thoughts, expectations and experiences of real-time access to test results in relation to their view of patient-centred care. Our research question was what patient discourses on real-time access to test results can be distinguished?

## Methods

### Study design

We conducted a microlevel discourse analysis to obtain a deep understanding of what real-time access means to patients.^30 31 35 36^ A discourse analysis uncovers how social reality is produced, unlike other qualitative methodologies which try to understand or interpret social reality as it exists.^30^ Discourse analysis also shows the problems and possibilities created by different discourses, allowing us to weigh their practical consequences.^34^ We conducted 28 semistructured interviews with patients who had or did not have experience with real-time access to test results on a patient portal. We conducted the first interview on 5 March 2018 and the last interview on 2 June 2021. This relatively lengthy data collection period reflects the time frame during which access to test results became increasingly available in the Netherlands.

### Patient and public involvement

The principal investigator (BP) of this study was involved in a national programme in the Netherlands in which patients articulated their struggles with real-time access to test results.^37^ Based on these results, this study was drafted. Patients helped to recruit patients for this study through the snowball method and thereby patients helped us find new participants for this study (also see next paragraph). We plan to disseminate the findings of this study via the Dutch Patient Federation.

### Participants and data collection

In the Netherlands, commercial information technology suppliers sell patient portals to Dutch hospitals. The hospitals can choose from suppliers for their patient portals, as long as the supplier complies with national standards (eg, MedMij).^38^ Our participants were treated in different hospitals so used different portals. These portals had different functionalities, but all offered online access to health-related information and test results.

The inclusion criteria were patients who (1) had visited an outpatient clinic in a Dutch hospital, (2) were 16 years or older and (3) had used or not used a patient portal to obtain real-time access to test results.

The researchers involved in this study are trained and experienced (AMJWMW-J and KA, BP) or were in training (PH and MB) in qualitative research. Moreover, the principal investigator of this research (BP) gained her PhD in generative discourse analysis.^39^ All researchers are well informed about patient participation in care processes and (healthcare) organisations.

We used purposive and snowball sampling to find participants. We aimed to select patients with and without experience of real-time access to test results. Three researchers (PH, MB and BP) began by approaching their own networks,^40^ calling potential participants to explain the study and inviting them to an interview. At the end of the interviews, we asked participants if they knew other patients we could interview (snowball method). In total, we included 28 participants (see online supplemental file 1, table 1 participant characteristics).

Aiming for a broadly inclusive participant sample,^31^ we included participants both with and without (never accessed) experience of real-time access to test results online. We felt it important to include diverse patients to understand the thoughts, expectations, needs and wishes of patients without experience. We began interviewing participants without experience in March 2018. At the time, few Dutch healthcare organisations offered real-time access to test results.^21^ We stopped interviewing when we reached data saturation (ie, new data no longer provided new insights) at which point we felt the sample of patients without experience was adequate.^41^ Two years later, more healthcare organisations offered real-time access, which in April 2020 enabled us to begin interviewing participants with experience. Again, when we reached data saturation in June 2021, we stopped interviewing. In total, we interviewed 13 participants without real-time access experience (8 females, 5 males) and 15 participants with real-time access experience (10 females, 5 males).

Two researchers (PH and MB) conducted 28 semistructured interviews with the participants according to a predefined topic list that was based on the literature outlined in the Introduction section. We developed two topic lists: one list for participants without real-time access experience asking about their expectations (see online supplemental file 2) and one list for participants with real-time access experience asking about their experiences (see online supplemental file 3).

The interviews were conducted at a participants’ preferred location or via video or phone during COVID-19 period. At the start of each interview, we introduced ourselves, repeated the information on the research aim, research methods and asked again for informed consent. The interviews lasted 45–60 min, were audio recorded and transcribed verbatim.

### Data analysis

We analysed data from participants with and without real-time access experience separately, as two data subsets, and checked for overlap and differences. We found no relevant differences between the two groups with regard to their discourses on real-time access to test results via a patient portal. Therefore, we continued to analyse the whole dataset as one. The analysis consisted of three steps.^35 42^ First, one researcher (PH) searched for the three entities that are constructed in a discourse: objects, concepts and subject positions.^35^ Objects are part of a practical order and exist in real life, such as the patient portal, healthcare professionals and patients in this context.^35^ In contrast, concepts exist only as ideas^35^ and show how patients construct the concept of real-time access to medical information on the patient portal and what they think about roles and responsibilities. Subject positions are assigned places in the interaction hierarchy and illustrate the power dynamics of relational processes (who was allowed to do what, who determined what and who did what).^35^ For example, think of a patient who is an expert on his or her own disease and who accesses their real-time available test results for self-management purposes, which in turn influences the role of the healthcare professional, namely like a coach.^29^ During this coding process, attention was paid to texts about responsibilities, rights and duties of both patients and healthcare professionals.

Second, two researchers (PH and BP) further coded these codes axially, including the advantages and disadvantages of real-time access described in the literature (see the ‘Introduction’ section) and the themes described by Pluut^29^ (eg, the themes for the discourse ‘caring for patients’ were ‘vulnerability’ and ‘healthcare professional decides on follow-up’). Any disagreements on codes were resolved by the researchers discussing them until consensus was reached.

Third, one researcher (PH) identified the most common codes for each theme by reading and analysing the transcripts and codes repeatedly. The coherence among themes was then analysed and discussed with three researchers (PH, AMJWMW-J and BP). This discussion led to a deeper understanding of the phenomenon under study as we critically examined our own assumptions on the topic during the discussion sessions. In this way, the ‘inter-related set of text and practices’ (p. 3, Parker, 1992)^30^ gave meaning to the discourses on real-time access.

## Results

We found three discourses on real-time access: (1) real-time access as a source of stress, (2) anxiety reduction through real-time access and (3) real-time access for self-management. Most participants talked from a dominant discourse, which means they explained their construction of real-time access to patient portals based on the main themes and arguments of one discourse. At the same time, patients can draw from different discourses when expressing their feelings, thoughts and dilemmas about real-time access to test results on patient portals.

### Discourse 1: real-time access as a source of stress

Real-time access as a source of stress frames real-time access as a potential stressor caused by various reasons. First, patients can feel stressed because of the ambivalence they have about the option of accessing their test results before the medical consultation or not. Within this discourse, patients both see the opportunities and risks, and find it difficult to predict whether reading their test results before the consultation will help them or cause more stress. Participants sometimes changed their minds on real-time access during the interview, or simply said they did not know what to do. This is illustrated in the following quotes from one participant, which show how their opinions changed as the interview progressed:

Well, I don’t know if I should check the results right away or if I should first wait for the conversation with the doctor. Then you see something, but then you don’t quite know what it is yet. […] [R1]

Yes, because that tumor in the bladder: it was huge, and you saw blood. That was all you saw. Of course, they [healthcare professionals], who took the image saw more. No, I think I would just wait for the physician. […] It doesn’t make me any wiser and I think it makes me more anxious. That I know the result and then I can do nothing with it. […] [R1]

Well, if it’s on there [read: the patient portal], I guess I’m curious enough to look anyway. I can’t really say that I wouldn’t. [R1]

Another participant who had experience with real-time access, but not with sensitive results, explained her doubts about the opportunities and risks of real-time access. She was quite relaxed about accessing some of her past results but did not know what to think about more sensitive results:

I can also read on how my results were a year before. It is not very exciting for me. If I’m waiting for a sensitive result, I would be nervous thus I am going to look at the results real-time or not? Then you want to know, but how are you going to read it? [R27]

The second stress source mentioned by our participants was the medical language use on patient portals. Within this discourse, medical language was constructed as a source of stress because participants were worried about interpreting texts incorrectly. One participant said:

It is good that test results are available, but as laypersons we are not directly aware how to interpret the results. The danger is that if you are in a long process—cancer or something—tumor marker, such a result can put someone on the wrong track. Those results could take on a life of their own. [R24]

During the interviews, participants commented that test results on the portal are not comprehensible enough. They said that it would be good to add some kind of explanation on the portal to avoid misinterpretation of the test results. One participant with experience in real-time access said:

Now, for example, if the result is too high or too low, it is not reported that these values can be interpreted differently. It would be nice if they [read: patients] get a little explanation in the portal and I don’t get that now. I’m smart enough to think about it carefully, but I understand that it can be a barrier for other people. [R18]

Participants also reported that it was quite easy to find information about severe diseases on the internet that could lead them to interpret their test results incorrectly, leading to unnecessary stress. One participant without experience in real-time access said:

Because then I think ‘oh I don’t know something’, so I look up (on the internet) what it is. But I do know that can be a big disadvantage. That you often find worse things. [R12]

The third source of stress was looking repeatedly on the portal to check if the results were already disclosed.

It has been said that there will be a result within ten days and then people will check it to see if the results are really on the portal within ten days. And that these people do not do anything with that result, but just check whether they are still receiving attention of the doctor. I get that idea from it. A friend told me about her father: every day I’ll check three times because the doctor said, ‘within 10 days the result will be on the portal’. So how much unrest can you have in your head for yourself? Looking so dramatically every time if the result is on the portal, how does that affect your life? [R19]

This quote illustrates how having access to test results can evoke stressful checking behaviour that can last for days until the test results are disclosed.

At the same time, patients who constructed real-time access as a source of stress did seem to appreciate the transparency in health information. One participant (who had not had experience accessing their results in real time) identified real-time access as ‘a good thing’ and said it was nice to be informed about their health, even though the doctor is considered to be the expert.

Real-time access is of course good; I am in favor that you can look at such a website and that you then see what is going on. Then you see what’s going on, but now imagine that there is something serious. At that moment you cannot ask [the doctor] what it is exactly. It’s nice that you can see the result when it’s good, but when it’s bad and you can’t have a conversation with the doctor […] I think you would like to know what the result is, so I think I will look. Only when it’s good, then you’re relieved. But if it’s not good news, then I think, maybe I shouldn’t have done that. But if you have a conversation with the doctor within a short time, they can give the necessary information. Then I would choose to look. [R8]

Some participants said they would prefer to get their results after their consultation to avoid stress caused by deciding whether or not to look at the portal, by not understanding medical terms, and by the danger of repeatedly checking the portal. These participants would rather use the test results disclosed on the portal as a record of what was discussed with their doctor.

Put the results on it [read: portal] if there has been an interpretation of the results in a consultation with the doctor. Then it is an addition to the consultation. [R24]

Participants also suggested ways to reduce the stress that comes from wondering whether to look at the test results before the medical consultation or not. They argued that informing patients about the advantages and disadvantages of real-time access to test results would help them decide whether to access their test results. They also suggested a conversation about real-time access with a professional could reduce stress. This plea is illustrated in the following citations from participants with experience in real-time access to test results:

If the hospital offers this kind of portal, they will also have an intention for a better patient experience or something like that. I also think it would be useful if the hospital informs their patients about this. Otherwise, you might as well not offer a portal. [R28]

Well maybe a conversation before the examinations start, like: ‘We have this portal, you can read all of this [on it]. Which do you prefer, that the result is discussed with you first or that it can be read immediately?’ [R19]

This discourse shows that participants appreciate transparency in health-related information. Within this discourse, participants framed the healthcare professional as the expert and the one with the medical knowledge. The disclosure of test results can cause stress for patients in three ways: (1) through the complex decision on whether or not to look at the test results before the medical consultation, (2) through the complex language use on the portal, which may cause misinterpretations, doubts and unclarity and (3) through the urge to repeatedly check if the results are already disclosed on the portal. This stress could be reduced by information on the advantages and disadvantages of real-time access and a conversation with the healthcare professional before using the portal. This would better inform patients on the choice they need to make about accessing their test results online.

### Discourse 2: anxiety reduction through real-time access

The first discourse constructed real-time access as a potential stress source. In contrast, this second discourse emphasises how real-time access may reduce anxiety. The anxiety reduction through real-time access discourse is based on the construction of a test being an emotionally charged event. Patients explain how they are very eager to know whether the result of a test is good or bad, and how real-time access can bring relief. This relief comes after the stress of waiting for a result that is constructed as important and impactful. Patients who centred the emotional aspects of accessing text results explained how they were aware of the possibility of bad and good news and how they hoped for the best. One participant, who had no experience with real-time access, said:

[…] You can be very relieved, but you can also have a big problem. So that can go both ways. If you’re worried about it and the results are not that bad, which will often happen, then that’s a relief. [R4]

In this discourse, patients reflected on how different tests can be more or less emotionally charged. The more worried they are, the more likely they are to access test results before the medical consultation. One participant articulated:

If they have done a breast puncture, for example, then I would like to know, because I can prepare myself: it will probably not be a nice conversation and what do I want to know from the specialist? With the Holder monitor it was about arrhythmias, and I wasn’t too worried. You can also think of a lot of scenarios, but then I think: I’ll hear that from the cardiologist. [R19]

Some participants compared looking at the test results with sitting an exam, where they have to wait for the result in suspense and, even if they are sensitive, would like to know. One participant without experience with real-time access said:

I would look at that moment because I’m curious. That’s like taking an exam, so to speak, if you know it’s going to be announced, even though you know you’ve done it badly, then you’re curious about how it is now. [R9]

Another patient without experience with real-time access said they believed it would reduce the stressful waiting:

I like real-time access because it gives you your results quicker. Usually, you must wait a few days for the results and now you don’t have to wait in stress, so I like that. [R11]

Other participants talked about how real-time access to test results would help them prepare for their consultation with the healthcare professional, even when getting the result is exciting. They also talked about asking somebody close to them to accompany them to the medical consultation:

I would like to see everything, yes. Because it’s about me. […] You already know that something is not right when you see those results. You see the deviating values, then you think okay, so apparently something is going on. So, prepare yourself for that. Then you can also think I’ll take someone with me during my outpatient visit, because two people always know more than one. [R7]

Within this discourse, patients seemed to accept that they would not always understand the results they read in the portal, but they also did not expect this to be a problem. Participants with and without experience with real-time access, were willing to ask their healthcare professionals or relatives with medical knowledge for help or use the internet to understand medical terms. To them, the temporary anxiety of not understanding was less problematic than the stress of not knowing at all and having to wait longer for the results:

If you know ‘it’s okay’, then I’m relieved. For example, if you don’t understand something, I think you can just call the assistant for more explanation […]. [R10]

Last week my sister asked me for help with interpreting her blood test results. She did not know what she should do with these results. Hence, I am able to interpret it. [R23]

[…] And even if you don’t fully understand the medical terms, well then I would just look it up. [R7]

Participants also mentioned that the disclaimer they read before receiving their test results was a good way of informing them that the information could be stressful and misunderstood.

You will then receive the disclaimer “with caution” which states the results do not say everything and discussion is needed with your physician before you panic. […] it’s fine that the warning is there. [R28]

Participants talked about how having information on their own health status made them feel responsible for discussing their results and the possible treatment/further action with their healthcare professional. The next citation illustrates the importance of discussing the results with the healthcare professional, who is framed as the expert with knowledge of health conditions and treatments:

Of course, I would have a look on the internet, but I would leave it to the doctor… then we can discuss together again, what can we do about it. [R10]

Although patients were willing to invest time and energy in finding out what the results they read in the portal mean and felt responsible for making decisions on follow-up treatment, they also said they would be appreciate it if healthcare professionals provided interpretations/reassurance in the patient portal to reduce stress:

I know, my general practitioner also releases results online. He always adds a comment first: ‘Don’t worry, nothing to worry about’. Something like that. Now [read: in the hospital portal], you miss that step. [R7]

In sum, this discourse constructed real-time disclosure as a means of reducing the anxiety that is inextricably linked to waiting for and mentally processing online test results. Participants were more likely to access results that were more emotionally charged before their hospital appointment. Anxiety reduction was especially important to participants, so they were willing to invest time and energy in understanding the information posted on the portal (eg, by asking medically trained friends/relatives or by searching on the internet). Participants also felt the need to be well informed about their health in order to make health-related decisions and discuss their results with the healthcare professional. Most participants who draw from this discourse perceive the healthcare professional as an expert with invaluable knowledge of health conditions and treatments. Therefore, participants suggested that healthcare professionals could explain test results to patients. This would help them understand their online results and improve their care.

### Discourse 3: real-time access for self-management

The real-time access for self-management discourse constructs real-time access as an important facilitator of self-management. Whereas the first two discourses centre the emotional aspects of real-time access to test results, this discourse focuses on the practical use of real-time access for self-management purposes and as something that makes the care processes more convenient. Within this discourse, test results are regularly checked to achieve various aims.

The first aim of regularly checking their test results was to become aware of their health status. One participant with experience of real-time access said:

I like real-time access very much because I can also read the results. What I like about my hospital is that everything is shown in graphs. No matter what test you open, you can always see how the blood values are rising, or blood platelets, urine tests, etc. […] I find that very pleasant. [R17]

The second aim of regularly checking the test results was to reflect on their health status and lifestyle. Two participants with experience of real-time access explained:

Sometimes, if you tell the doctor ‘I am extremely tired’. This could be an iron deficiency. Then I have a blood test and I can immediately see whether my iron level is too low. Then you have confirmation that your assumption is correct. So, I like that. It is also a reassurance of good numbers. [R18]

I recently had a visit to the hospital and we [read: patient and patients’ partner] are both curious. I know I can check after one or two days. Then I know and then it’s well. I know at that moment; I’m doing the right thing. [R27]

The third aim of regularly checking their test results was to make the medical consultation more substantive by better preparing them for the consultation. Patients asked more specific questions if they had looked at their test results before the medical consultation. They also felt that they could respond more critically to the physician’s explanation of their test results.

I like that you immediately can benchmark your reference values, you don’t know a lot of those numbers exactly. The most pleasant values are the ones a bit near the limit or just below, especially relevant to ask questions about. My physician tends to say that everything is going well. I believe that too, but it is nice that you have a little more information and are enabled to ask them questions about the values. [R28]

The fourth aim of regularly checking their test results was to put them more in charge of the conversation with their healthcare professionals during the medical consultation. One participant with experience of real-time access said:

I like real-time access because you don’t go into the conversation unprepared. I speak to the internist once every six months, three times a year and then you get the results, and I would like to know in advance whether things are going better. Whether it [read: the result] is more stable. So, I can look into that […] I think the internist knows I’m looking at my results before our conversation, but we’ve never discussed it so emphatically. [R27]

The fifth aim of regularly checking their test results was to be able to immediately act on the results. This enables patients to obtain quicker treatment and increase their safety by, for example, calling the doctor and asking questions about the test results on the patient portal:

I read the report: ‘There was nothing unusual in the blood results.’ Then I looked at the results and then I saw his [read: my father] hemoglobin (HB) level is much too low and he has high inflammation values. So, I called the doctor and said: ‘I don’t want to be a smartass, but when I look at the lab results, I see that the HB level is quite low, and the inflammation values are high. Is he on medication for that?’ The doctor said, ‘I’ll have to check that’. Half an hour later he called and said: ‘Good that you checked it, indeed he must have medication for that’. Thus, it [read: online access] can also go in the right direction. I found that very striking. I hardly ever look at the results in the portal. But at the time, I was really glad I looked at it. [R19]

Furthermore, participants constructed another reason for finding real-time access an aspect of good care: their body and health. One participant without experience of real-time access said:

I think real-time access is a very good development. Why not? It’s about yourself, right. [R7]

In addition, participants commented on the importance of transparency in health-related information for their own decision-making. The participants framed patients’ responsibility in the decision-making process and the right to know their own health-related information. One participant without experience in real-time access stated:

I’m positive about it [read: real-time access]. I think that you should also have knowledge of your own medical file. What is known by the doctor and the nurse et cetera, I think that is at least what I should know. [R9]

Some participants constructed themselves as an expert of their own health and felt that the healthcare professional could not add much new information. This was especially true for patients with a chronic disease:

I think I look up or know most of it myself. I am also a member of the diabetes association and then you also receive magazines and newsletters. She [read: nurse] can’t really add anything more. The hospital also has information leaflets and things like that. But when I need information, I look it up. [R28]

Participants become an expert based on the comprehensibility of their test results, which was framed as a learning process. These participants saw their physician as a coach or guide, who explained their test results. For example, one participant with experience of real-time access who checked his ignition values learnt over time how he should interpret these values.

I’ll check the ignition values. I got an explanation from a doctor once: ‘The inflammation value is high, but you must see it in relation to that other value, it is low again, so in the end it’s not too bad’. You cannot interpret that yourself if you don’t have this information. [R25]

As part of the learning process, participants constructed themselves as being able to easily look up medical terminology they are not familiar with on the internet. One participant with experience of real-time access said:

I am always someone who likes to look up everything, because sometimes my healthcare professional has requested to test things, and those [read: test] are far too difficult words and then I will look it up myself to understand ‘Gosh what does that mean’. But there is no explanation or anything on the portal. [R18]

Participants also articulated that, for convenience, they wanted a check mark on the portal that showed whether the doctor had already looked at their test results or not.

I don’t see a check mark: the doctor has reviewed and assessed and will contact you if treatment is needed. [R25]

Besides the check mark for convenience, participants also said that they would like to be able to check what is in their patient file and, if necessary, correct any mistakes. They considered it their responsibility to correct this information and look at their test results soon as they are available. Participants mentioned that their whole patient file is not on the portal. One participant with experience in real-time access explained:

I have the feeling that there is a lot being written that I don’t see on the portal. The patient portal provides a kind of insight, on I don’t know what. My new doctor didn’t get a file from me, other than a few lines of information. Although I think they have written quite a lot about me. I remember that the diabetes nurse had used ‘motivational conversation’ methodology to discuss my disease. They also point out things about me that I don’t think are right. What kind of image emerges about who I am? [R26]

Within this discourse, participants had five aims for regularly checking their test results: (1) to be aware of their health status, (2) to reflect on their health status and lifestyle, (3) to prepare questions for their medical consultation, (4) to be in charge of the conversation during the medical consultation and (5) to act in response to their test results in order to speed up treatment and increase safety. In this discourse, patients are seen as the experts of their own health and real-time access to test results is seen as a natural part of the care process for optimal self-management and convenient care. Healthcare professionals were seen as coaches or guides who explained incomprehensible medical language. Participants also discussed how they cannot completely fulfil their responsibilities because they do not know whether the doctor has looked at their test results (no check mark) and because their entire medical record is not available on the patient portal (incomplete transparency). Participants framed these omissions as missed opportunities for delivering good care with real-time access to test results on patient portals.

## Discussion

### Principal findings

This study aimed to provide a deep understanding of patient discourses on real-time access to test results on a patient portal. We considered the practical consequences of various discourses^33 34^ for (1) policy development by healthcare providers, highlighting the possible implications of policy options and (2) the design of technologies, healthcare processes and information to help patients and healthcare professionals make decisions about real-time access. Our research question was what patient discourses on real-time access to test results can be distinguished? We identified three discourses: (1) source of stress, (2) anxiety reduction and (3) self-management.

Within these discourses, we identified two recurring themes constructed differently in each discourse. The first theme, coming from the patient’s perspective concerned the complex language and jargon used in test results. The first discourse, source of stress highlights the risk of misunderstanding information and the stress that can arise from searching the internet for information to help understand and interpret test results. The second discourse, anxiety reduction emphasised the patient’s ability and willingness to ask people with medical knowledge for help when reading complex information or to search for an explanation on the internet. The third discourse, self-management framed handling complex language as a learning process, where patients can empower themselves by increasing their knowledge and learning where to look and what to search for on the internet.

The second recurring theme arising in all discourses was the value of transparency, which was also linked to other values and elements of patient-centred care. Source of stress emphasised the value of patients being well-informed, no matter if the information was provided before or after the consultation. Anxiety reduction related the value of well-informed patients to the emotional relief of knowing the result of a test as soon as possible. Interestingly, self-management also linked the value of patient empowerment to the value of transparency because portal information offered self-management opportunities.

### Practical implications

Each discourse has practical consequences for healthcare organisations’ policies on real-time access to test results and the ways in which they can embed it into daily practices. Source of stress highlighted the importance of reducing possible emotional damage by informing patients about real-time access. A recent study^43^ found no link between precounselling and reduced patient worry levels, possibly due to the focus on explaining the testing rationale. Precounselling could incorporate both technical and sociotechnical methods.^43^ This discourse suggests that providing real-time access to information can be done in three ways.

First, we showed that patients want to be informed about the advantages and disadvantages of real-time access. Earlier research has shown that patients are not always informed about patient portals^2544^,^46^ and do not know that they can access their test results before the medical consultation. A recent study confirms that patients should be informed about patient portals in general, and specifically the pros and cons of using them.^47^ This has implications for portal design as it should offer explanatory texts or videos for users.

Second, we showed that giving patients real-time access can evoke stressful checking behaviour that can last for days until the test results are disclosed. To avoid this, portals could notify patients when their results are published. One study suggests two notification policies: immediate notifications for all results and only for patients who have opted-in for notifications.^48^ In this case, however, healthcare providers must uphold promises to deliver test results in real time.

Third, we showed that patients want their healthcare professional’s advice on whether or not to access their test results before the consultation. This is in line with the findings of other studies, which conclude that healthcare professionals should anticipate what patients might see at the portal,^11^ discuss whether real-time access is a good idea, be available to answer questions^15^ and have a transparent discussion on the patient’s notification preferences for abnormal test results.^49^ Further research should focus on how to support healthcare professionals and patients in this shared decision-making process.

Both sources of stress and anxiety reduction show that healthcare providers and healthcare professionals need to think about how patients might interpret test results to avoid misinterpretation. We showed that patients want comprehensible explanations of their test results. This is in line with an earlier qualitative study showing that reference values for test results and doctor’s comments helped patients to understand test results on the portal.^50^ Also, two other studies demonstrated how the doctor’s interpretation alleviated patient anxiety.^11 14^ In a recent study, patients recommended other options, such as a glossary of terms for complex medical results, supplementary follow-up information, and layman’s summaries of reports to enhance test result interpretation.^51^ This suggests that patient portals should incorporate reference values for test results, health-related information in layman’s terms and open notes from healthcare professionals. Even with reference values displayed, patients want confirmation from healthcare professionals on the accuracy of their interpretation of test results.^52^ This also implies that the healthcare professional should give an oral explanation of how to interpret test results is a necessary part of a medical consultation. Healthcare professionals and communication advisers could help portal developers provide the necessary explanations in layman’s terms on the portal.

Self-management highlighted possible ways to broaden the functions of patient portals. First, we showed that patients want to know if the doctor has checked their test results, as a study on real-time access to oncology results also reports.^51^ Designers of patient portals could consider adding a check mark that lets patients see if the healthcare professional has seen and approved their results. This means healthcare professionals may have to adjust their work processes based on when and how they check results. We also showed that patients want to correct inaccuracies and want more transparent health-related information in their medical files, in line with the findings of earlier studies.^7 53^

Healthcare organisations can involve patients in the implementation, integration and evaluation of policies related to real-time access to test results. The evaluation can take the form of action research,^54^ where patients and professionals cocreate, evaluate and improve the processes around access to test results during the research process.

### Limitations

This study has limitations. First, the snowball method we used, starting with our own network may have caused bias.^55^ Our sample does not represent all ages as the age categories 31–45 and 76–90 are under-represented (see online supplemental file 1, table 1). Therefore, our results should be generalised with caution. However, the 28 people we interviewed provided a good overview of opinions and experiences on real-time access to test results. Second, participants often mentioned ‘other people’ instead of their own experiences. This may affect the validity of our study because assumptions of others’ experiences do not always match what is actually experienced.^41^ Speaking of others might also indicate that participants were uncertain of real-time access because they might not have had enough information and were still forming an opinion.

Third, we did not stratify our sample, which could account for the differences among discourses according to gender or type of result (sensitive/not sensitive, routine/diagnostic). Earlier studies on result types and real-time access have mixed findings.^20 56^ For instance, in a study of 30 patients with cancer, accessing laboratory results in real-time reduced anxiety.^20^ Another study reported most patients preferred real-time access for less sensitive diagnoses (high cholesterol, strep throat, genetic disease, sexually transmitted disease) but preferred a time delay for sensitive results (Alzheimer’s disease, fetal miscarriage, cancer).^56^ Please note, however, that our microlevel discourse analysis aimed to describe discourses, not to explain them. We wanted to examine how individuals socially construct a new technological functionality, namely real-time access to test results via patient portals. Articulating the differences in the social constructs of different discourses invites us to reflect on the practical implications of each discourse. This in turn could inform the design and embedding process of patient portals. Further research could expand or enrich the three discourses by zooming in on the differences in demographics, such as type of test result, age and sensitivity of the result. For example, one scoping review showed that older patients, those unfamiliar with portals, and those with abnormal results or conditions like cancer, cardiovascular disease or depression use portals less often for radiology results and prefer direct communication with a physician.^11^ These patients are likely to frame real-time access as a source of stress.

Fourth, we conducted this study before and during the COVID-19 pandemic. Patient perspectives on receiving test results via the patient portal may have shifted in response to the new and/or temporary online practices that emerged during the pandemic.

### Comparison to prior work

Our findings confirm previously observed advantages of real-time access for patients. These include (1) processing their test results better at home and being better prepared for the consultation with healthcare professionals,^1820^,^22^ (2) developing a strong sense of ownership of their results^12 43^ and being better informed^12 14 23^ and (3) increasing their own safety by checking their results and responding quickly to abnormalities.^14^ Our findings also confirm previously observed disadvantages of real-time access. These include difficulties with interpreting test results,^12^,^1423^ sometimes causing unnecessary anxiety,^12 14 15 23^ stress^22 42^ and confusion.^15^

We found two disadvantages, not identified in earlier studies: (1) being faced with the hard decision of whether to look at the test results before the medical consultation caused stress and (2) repeatedly checking if results were available evoked stressful checking behaviour which could last for days until the test results were disclosed.

We also derived new implications for portal design and healthcare processes given the different constructs patients have for real-time access to test results on a patient portal. We can account for these differences by looking at their constructs of ‘good care’. All participants seemed to value transparency in health-related information, which agrees with the findings of Leonard *et al*.^57^ Still, not all patients considered real-time access to test results as ‘good care’. Some constructed themselves as vulnerable and believed it is the healthcare professional’s responsibility to care for them.^29^ These patients were more likely to emphasise the emotional aspects of real-time access, such as causing stress or reducing anxiety. Patients who emphasised their own responsibility for ‘good care’ were more likely to focus on the practical opportunities, such as self-management.^29^ A recent study on information transparency through real-time access in oncology suggests a shift in medical decision-making from a paternalistic to a patient-centred approach. This implies that some professionals believe that if given information patients can make informed decisions and thus actively participate in their own care.^51^ Real-time access for self-management may increasingly be viewed as exemplary care. Another article^47^ underscores that real-time access to their test results and medical file empowers patients in health decision-making. However, our study reveals a diversity of patient expectations and experiences regarding real-time access, suggesting that self-management opportunities represent just one aspect of ‘good care’.

## Conclusions

Our study provides in-depth insights and highlights practical implications for various stakeholders, such as policy-makers and eHealth technology developers. The discourse analysis showed the plurality in patient expectations and experiences. We found three discourses (see online supplemental file 4, table 2 an overview of patient discourses on real-time access to test results) that illustrate the different ways in which real-time access can be constructed and how healthcare providers and patients can make optimal use of real-time access to test results on patient portals from a patient perspective.

## supplementary material

10.1136/bmjopen-2024-088201 online supplemental file 1

10.1136/bmjopen-2024-088201 online supplemental file 2

10.1136/bmjopen-2024-088201 online supplemental file 3

10.1136/bmjopen-2024-088201 online supplemental file 4

## Data Availability

Data are available on reasonable request.
