# Supplementary material for: Patient discourses on real-time access to test results via hospital portals: a discourse analysis of semistructured interviews with Dutch patients
Source: BMJ Open. 2024 Nov 24;14(11):e088201. doi: 10.1136/bmjopen-2024-088201 (PMC11590850; doi:10.1136/bmjopen-2024-088201)
Supplement: online supplemental file 1 [file bmjopen-14-11-s001.docx]

**Supplemental file 1.** Table 1. Participant characteristics.

| **Age (years)** | **Participants (N=28)** | | | |
| --- | --- | --- | --- | --- |
|  | **Female (n=18, 64%)** | | **Male (n=10, 36%)** | |
|  | **Experience with real-time access (n=10, 56%)** | **No experience with real-time access (n=8, 44%)** | **Experience with real-time access (n=5, 50%)** | **No experience with real-time access (n=5, 50%)** |
| **16–30** | 2 (20%) | 4 (50%) | 2 (40%) | 1 (20%) |
| **31–45** | 2 (20%) | 0 (0%) | 1 (20%) | 0 (0%) |
| **46–60** | 3 (30%) | 3 (37.5%) | 0 (0%) | 1 (20%) |
| **61–75** | 3 (30%) | 1 (12.5%) | 2 (40%) | 2 (40%) |
| **76–90** | 0 (0%) | 0 (0%) | 0 (0%) | 1 (20%) |
