# Supplementary material for: Patient discourses on real-time access to test results via hospital portals: a discourse analysis of semistructured interviews with Dutch patients
Source: BMJ Open. 2024 Nov 24;14(11):e088201. doi: 10.1136/bmjopen-2024-088201 (PMC11590850; doi:10.1136/bmjopen-2024-088201)
Supplement: online supplemental file 2 [file bmjopen-14-11-s002.doc]

## Supplemental file 2: Topic list for participants without real-time access experiences

|  | Questions |
| --- | --- |
| Opening interview | First, I will explain what our study is about. We have just discussed that you, (NAME), give permission for this interview to be recorded and that the data will be used anonymously. The data will only be used for this research. Agree? |
| Facts (appoint gradually during the interview) | Gender  Age  Do you use a phone, computer, or tablet? For what do you use it for?  Do you use e-mail? |
| Good care | First of all, I am very curious about how you feel about good healthcare?  When was the last time you visited a hospital?  May I ask which outpatient clinic you visited?  Looking back on that visit, what did you like about the care?  How was the conversation with the doctor?  Can you give an example of things that you think were not right at that moment?  You mention X and Y as starting points for good care – are there any more?  A & B did not go well, this means that you value A & B regarding good care?  When you visit the doctor at the outpatient clinic, what is the ideal conversation with the doctor?  How is the treatment you receive from the doctor?  Does the doctor listen to you carefully? How do you know?  Do you think your doctor is an expert?  Do you think I have a good idea of what you think is good care? |
| Patient-centeredness | What else do you consider important in your relationship with your doctor? And further?  Can you give an example of what you think is patient-centered?  Do you think I have a good idea of what you think is patient-oriented? |
| Patient image | When do you consider yourself as a patient?  When do you see someone else, for example a friend or neighbour, as a patient?  So when we look at when you consider yourself or someone else as a patient, we can conclude that someone is a patient if ... .. (and then let the participant fill this in)  A distinction is often made between active patients and passive patients. How do you see yourself?  Most people would agree that both the doctor and patient affect the quality of care. What do you think is the responsibility of the patient?  Consider your last visit to the hospital. How did you influence the quality of care? What did you think was your responsibility in this?  Do you think I have a good idea of your opinion of when you consider yourself a patient? |
| Most important task of a patient | What do you think is the most important responsibility of the doctor?  What is important to you when you visit the doctor in the outpatient clinic from the hospital?  How is the treatment provided by the doctor? Do you think that is important?  How is the information provided by the doctor? Do you think that is important?  How is the doctor's decision-making? To what extent are you involved?  Do you think I know what you think the doctor’s most important task is? |
| Responsibility for decision-making | Who should make decisions about your treatment?  Thinking back to your hospital visit, can you give an example of where a decision had to be made about your therapy?  How was it decided, which treatment you received?  To what extent did you determine together with the doctor which care you received?  To what extent have your wishes been taken into account when choosing the therapy?  Do you think I have a good idea of your opinion on decision-making in treatment? |
| Information | What do you find important about the information you receive?  Suppose you have to go to hospital for a day for treatment. What do you do with the information you receive in advance?  How would you prefer to receive the information?  What do you think is important to see on a website, where you can view data on your own?  Suppose you have a chronic illness and therefore have to regularly visit the hospital laboratory for blood tests. Your treatment depends on the results of the blood test. Now it appears that you can see the blood results at the same time as your doctor, before you meet the doctor. What would you do if you could see all of your blood test results before speaking with the doctor?  Do you think I have a good idea of what you find important in the information you receive? |
| Real-time access through a patient portal | What do you think of real-time access to test results via a patient portal?  Imagine you have visited the hospital and you have had several tests. The results of those tests will be announced within four business days and you will have an appointment seven business days later with your doctor. What do you think about being able to see the test results at home before you have spoken to your doctor?  What would you do?  Nictiz, an organization with a lot of knowledge about eHealth, has listed the advantages and disadvantages of direct access to test results. I would like your opinion on these points.  An advantage according to Nictiz is that direct access ensures that  patients are not unnecessarily stressed and can process the results as soon as they are known. What do you think about this?  Another benefit that is mentioned is that patients can immediately  act based on the results. What do you think about this?  Patients can decide themselves whether they want to see the results immediately or not.  In case of a bad result, patients can process this in their own environment and are therefore better able to enter into a conversation with their doctor to discuss treatment positions. So, for example, a result about whether you have anemia or cancer. How do you see this?  These were the benefits mentioned by Nictiz, do you think there are more advantages of directly viewing data through a website?  A disadvantage of looking directly at the test results before talking to the doctor is that there is a greater chance of misinterpreting the result. This can lead to stress and possible disappointment. How do you see this?  Another disadvantage is that patients see information even though they have the right to not know. You also have the right not to know something, but it can be difficult to ignore curiosity. What do you think about this?  Finally, another disadvantage is that patients may contact the hospital because they have questions about their results. And that takes time for the hospital. What do you think about this? Would you do this yourself?  These were some of the disadvantages of directly accessing test results via the website, do you think there are any other disadvantages?  Now that you've heard all these advantages and disadvantages, I'm asking again for your answers to the following questions:  Imagine you have visited the hospital and you have had several tests. The results of those tests will be announced within four business days and you will have an appointment with your doctor in seven business days. What do you think about being able to see the test results at home before you have spoken to your doctor? What would you do?  Do you think I have a good idea of your opinion on direct access to test results? |
| Closing | Do you want to add or emphasize something?  Thank you very much for the interview. |
