# Supplementary material for: Patient discourses on real-time access to test results via hospital portals: a discourse analysis of semistructured interviews with Dutch patients
Source: BMJ Open. 2024 Nov 24;14(11):e088201. doi: 10.1136/bmjopen-2024-088201 (PMC11590850; doi:10.1136/bmjopen-2024-088201)
Supplement: online supplemental file 4 [file bmjopen-14-11-s004.docx]

**Supplemental file 4. Table 2. An overview of patient discourses on real-time access to test results.**

|  | **Discourse 1: *Real-time access as a source of stress*** | **Discourse 2: *Anxiety reduction through real-time access*** | **Discourse 3: *Real-time access for self-management*** |
| --- | --- | --- | --- |
| **Key words** | Stress, complexity, and ambivalence. | Stressful waiting period, preparation, and relief. | Facilitator of self-management, frequent checking. |
| **Implications of complex language use** | Ambivalence about whether to look at test results and risk of misunderstanding and stress due to searching on the internet. | Willingness to ask healthcare professionals or relatives with medical knowledge for explanation or search for an explanation on the internet. | Learning process: patients can and will learn where to look and what to search for on the internet. |
| **What is the value of real-time access to test results?** | The value of transparency.  The value of being well-informed as a patient before or after a medical consultation. | The value of transparency.  The value of being well-informed as a patient.  The value of emotional relief. | The value of transparency.  The value of being empowered as a patient. |
| **How to provide patient-centered real-time access to test results on a portal? (practical consequences)** | Reducing stress by providing information about the advantages and disadvantages of real-time access, also on the patient portal by texts or videos, and offering patients a conversation with a healthcare professional (pre-counseling). | Reducing anxiety by providing reference values for test results on the portal, health-related information in layman’s terms as well as open notes from the healthcare professionals and providing comprehensible explanations on interpretation of test results from healthcare professionals. | Improving self-management opportunities by providing a check mark on the portal that indicates whether the doctor has looked at the test results, more transparency on health-related information in the medical file, and the possibility for patients to correct inaccuracies in their file. |
