# Supplementary material for: Patient discourses on real-time access to test results via hospital portals: a discourse analysis of semistructured interviews with Dutch patients
Source: BMJ Open. 2024 Nov 24;14(11):e088201. doi: 10.1136/bmjopen-2024-088201 (PMC11590850; doi:10.1136/bmjopen-2024-088201)
Supplement: Supplementary file 5 [file bmjopen-2024-088201.reviewer_comments.pdf]

PEER REVIEW HISTORY

BMJ Open publishes all reviews undertaken for accepted manuscripts. Reviewers are asked to complete a checklist review form and are provided with free text boxes to elaborate on their assessment. These free text comments are reproduced below.

ARTICLE DETAILS

Title (Provisional)

Patient discourses on real-time access to test results via hospital portals: a discourse analysis of semi-structured interviews with Dutch patients

Authors

Hulter, Pauline; Weggelaar-Jansen, Anne Marie JWM; Ahaus, Kees; Pluut, Bettine

---

VERSION 1 - REVIEW

Reviewer 1

Name Chudner, Irit

Affiliation College of Management Academic Studies School of Business Administration, Bussineaa Administration

Date 02-Aug-2024

COI no competing interests

	Comment
General	This research focuses on real-time access to test results via patient portals, aiming to understand patients' decision-making processes regarding viewing results before meeting with their doctor. The researchers identified three themes: Real-time access as a source of stress, Anxiety reduction through real-time access and Real-time access for self-management.<br><br>The research is interesting and well written and addresses a relevant and current topic in digital health.<br><br>The use of discourse analysis allows for a deep understanding of patients' different viewpoints. The sample includes patients with and without experience, enabling an interesting qualitative comparison and the themes provide insightful perspectives on the advantages and disadvantages of real-time access.<br><br>The Abstract is well structured and clear.
Introduction	The introduction flows logically from defining patient portals to discussing the specific issue of real-time access to test results. This introduction

	provides balanced perspective through presentation of both advantages and disadvantages of real-time access,<br><br>The content is backed by citations, shoing a solid foundation in existing literature.<br><br>research gap clearly described by authors highlighting the lack of qualitative research on patients' experiences and decision-making processes.<br><br>Some suggestions to be considered by authors: Some parts could be more concise. For example, the definition of patient portals maybe could be shortened without losing essential information. The content of the table 1 can be seamlessly integrated into the text to create better flow. The introduction also could benefit from a more explicit statement of the research question or objective at the end.<br><br>In addition, there are several types of discourse analysis methods, each with its own focus and approach, which one did you used?
Methods	The choice of discourse analysis is well-justified, explaining how it can reveal patients' perspectives in the context of their lived experiences. Description of the discourse analysis approach, participant selection, data collection, and analysis process is well described.<br><br>Good participant diversity including both experienced and inexperienced users of real-time access<br><br>28 participants is a reasonable number for qualitative research, but a brief explanation of how this sample size was determined would be beneficial.<br><br>Mention whether data saturation was reached and how this was determined.<br><br>how disagreements between coders were resolved?<br><br>Timeline clarity: The data collection period (March 2018 to June 2021) is quite long. Please explain why this extended period was necessary
Results	The results section includes relevant quotes that effectively illustrate the themes and subthemes of the discourse. Suggestions from participants on how to mitigate stress, which is valuable for practical application of the findings
Discussion	The discussion effectively summarizes the main findings and links them to the research question and provides specific recommendations for healthcare providers, policy makers, and technology designers. The findings are well-contextualized within the existing body of research, highlighting both confirmations and new insights.<br><br>Some suggestions: The discussion is quite long and could potentially be condensed for improved readability.

	More discussion on how the findings might differ across demographic groups could be valuable.<br><br>Consider discussing how patients could be involved in implementing the recommendations derived from this stud

---

Reviewer 2

Name Steitz, Bryan D

Affiliation Department of Biomedical Informatics, Vanderbilt University Medical Center, Nashville, Tennessee.

Date 30-Aug-2024

COI I have no competing interests to report.

Thank you for the opportunity to review this manuscript. The manuscript reports a qualitative study of patient perspectives towards receiving test results via patient portal in The Netherlands. This study topic is timely and relevant to the journal readership. As written, there are several opportunities for improvement.

My primary concern is that the focus of the study, test results, does not provide enough context or nuance to fully understand the various patient attitudes and perceptions around the breadth of test results and clinical contexts. Prior research has shown that patient attitudes towards reviewing test results vary based on the type of result (e.g. lab test versus imaging), result sensitivity, prior discussion about the result and/or possible outcomes (e.g. anticipatory guidance), patient characteristics (e.g. attained education, existing health utilization), and numerous other factors. Without this context, it is presently difficult to contextualize the presented results with other, related, studies.

Additionally:

In the Abstract, the Participants section suggests that only patients without experience to result review were interviewed, but the Setting section and manuscript conflict this. Please clarify whether patients who received test results were included in the study.

In the second paragraph of the Introduction, the authors describe that providers can choose the time horizon for which test results are released. Please clarify that requirements for result release vary by country, and that the stated release strategy is specific to The Netherlands.

Throughout the manuscript, the authors refer to “(dis)advantages” around accessing test results. It is unclear whether this refers to advantages and disadvantages, or only disadvantages. Please clarify throughout the text.

Table 2 presents participant characteristics including sex, age, and experience with portal. However, given the numerous patient characteristics that may influence perceptions about result review, it would be beneficial to expand this table to further describe the study population. Other possible metrics may include attained education, health utilization, and existing technology use.

Page 10, line 5: The inclusion criteria that patients "had or had not used a patient portal.." reads as irrelevant given that this criteria encompasses both possible options. Consider removing for clarity.

On page 13, line 27: This first sentence is redundant and should be revised for clarity.

Throughout the Results, the authors refer generally to the number of participants who responded according to a particular theme. For example, on page 13, line 44 mentions "…participants were worried about interpreting…". Please consider quantifying the number of patients who responded according to the stated themes.

On page 30, line 46: Reference 41 appears to refer to a study about patient perceptions of receiving immediately released test results. This study does not present empiric data about patient notification preference as cited in the manuscript text. I suggest the following reference instead: "Impact of notification policy on patient-before-clinician review of immediately released test results"; https://doi.org/10.1093/jamia/ocad126.

Since this study was conducted before, during, and after the COVID-19 pandemic, the authors should include the possibility that patient perspectives about receiving test results via patient portal may have changed in response to new or temporary practice patterns caused by the pandemic.

VERSION 1 - AUTHOR RESPONSE

Dear Dr. Irit Chudner (reviewer 1),

Thank you for reviewing our paper and providing very helpful comments to improve our paper. Here is our point-by-point response to your comments.

General

This research focuses on real-time access to test results via patient portals, aiming to understand patients' decision-making processes regarding viewing results before meeting with their doctor. The researchers identified three themes: Real-time access as a source of stress, Anxiety reduction through real-time access and Real-time access for self-management. The research is interesting and well written and addresses a relevant and current topic in digital health. The use of discourse analysis allows for a deep understanding of patients' different viewpoints. The sample includes patients with and without experience, enabling an interesting qualitative

comparison and the themes provide insightful perspectives on the advantages and disadvantages of realtime access. The Abstract is well structured and clear.

Thank you for your compliments.

Introduction

The introduction flows logically from defining patient portals to discussing the specific issue of real-time access to test results. This introduction provides balanced perspective through presentation of both advantages and disadvantages of real-time access, The content is backed by citations, shoing a solid foundation in existing literature. research gap clearly described by authors highlighting the lack of qualitative research on patients' experiences and decision-making processes.

Some suggestions to be considered by authors: Some parts could be more concise. For example, the definition of patient portals maybe could be shortened without losing essential information. The content of the table 1 can be seamlessly integrated into the text to create better flow. The introduction also could benefit from a more explicit statement of the research question or objective at the end. In addition, there are several types of discourse analysis methods, each with its own focus and approach, which one did you used?

Thank you for your compliments and useful suggestions on making the Introduction more concise.

We shortened the definition of patient portals:

> "Patient portals can be defined as "*provider-tethered applications that allow patients to access, but not control, certain health care information (e.g., their electronic health record) and provide communication and administrative functions [...]*" (p.2) [1]."

We integrated information from the table into the text to improve the flow, as follows:

> The literature identified three advantages. First, patients are better able to process their test results at home and prepare for the outpatient consultations with their healthcare professional [18,20-22], e.g., by preparing questions [14,21]. Second, patients develop a strong sense of ownership in relation to their results because they can decide for themselves when to access them [12] and become more informed prior to the medical consultation [12,14,23]. This enhances shared decision-making with their healthcare professional [13]. Third, patients can review their results and contact their healthcare professional earlier if their results are abnormal, which enhances patient safety [12]. A disadvantage could be that patients may find it difficult to interpret their test results [12-14,24] potentially leading to (unnecessary) anxiety [12,14,15,23,25], stress [22], and confusion [15].

> These advantages and disadvantages signal that deciding when to disclose test results is not a simple technological choice made by the healthcare provider and/or the patient.

In the final paragraph of the Introduction, we explicitly mention the research objective and question:

> Besides investigating practical consequences, the aim of our study was to obtain a deeper understanding of patients' feelings, thoughts, expectations and experiences of real-time access to test results in relation to their view of patient-centered care. Our research question was: What patient discourses on real-time access to test results can we distinguish?

The Study Design (Method section) mentions a microlevel discourse analysis, which is now also mentioned in the Introduction:

> This microlevel discourse analysis gave us a deeper understanding of the feelings, thoughts, expectations and experiences of real-time access to test results from a patient perspective.

Method

The choice of discourse analysis is well-justified, explaining how it can reveal patients' perspectives in the context of their lived experiences. Description of the discourse analysis approach, participant selection, data collection, and analysis process is well described. Good participant diversity including both experienced and inexperienced users of real-time access 28 participants is a reasonable number for qualitative research, but a brief explanation of how this sample size was determined would be beneficial. Mention whether data saturation was reached and how this was determined. how disagreements between coders were resolved? Timeline clarity: The data collection period (March 2018 to June 2021) is quite long. Please explain why this extended period was necessary

Thank you for your compliments and suggestions. We tried to design a sample that was not only as broad and inclusive as possible [31] but also balanced, by including participants both without and with experience of real-time access to test results. This type of research has no specific answer to the question of determining sample size [41]. We stopped interviewing on reaching data saturation, at which point we considered the sample size adequate [41]. We did this twice, first for participants without experience, and second for participants with experience of real-time access to test results.

References:

31. *Wetherell M, Taylor S, and Yates SJ. Discourse as Data. A guide for analysis. The Open University: Walton Hall. 2010. ISBN: 0-7619-7158-0*

41. *Cresswell JW, Cresswel, JD. Research Design. Qualitative, quantitative & mixed methods approaches. Los Angeles, London, New Delhi, Singapore, Washington DC, Melbourne: SAGE publications. 2018. ISBN: 9781506386768*

The text under ‘Participants and data collection’ now reads:

> Aiming for a broadly inclusive participant sample [31] we included participants both with and without (never accessed) experience of real-time access to test results online. We felt it important to include diverse patients to understand the thoughts, expectations, needs, and wishes of patients without experience. We began interviewing participants without experience in March 2018. At the time, few Dutch healthcare organizations offered real-time access to test results [21]. We stopped interviewing when we reached data saturation (i.e., new data no longer provided new insights) at which point we felt the sample of patients without experience was adequate [41]. Two years later, more healthcare organizations offered real-time access, which in April 2020 enabled us to begin interviewing participants with experience. Again, when we reached data saturation in June 2021, we stopped interviewing. In total, we interviewed 13 participants without real-time access experience (8 females, 5 males) and 15 participants with real-time access experience (10 females, 5 males).

Any disagreements between coders were resolved by discussion until consensus was reached. This point is added under ‘Data analysis’:

> Second, two researchers (PH, BP) further coded these codes axially, including the advantages and disadvantages of real-time access described in the literature (see Introduction) and the themes described by Pluut [29] (e.g., themes for the discourse ‘caring for patients’ were ‘vulnerability’ and ‘healthcare professional decides on follow-up’). Any disagreements on codes were resolved by the researchers discussing them until consensus was reached.

We have clarified the data collection period:

> We conducted the first interview on 5 March 2018 and the last interview on 2 June 2021. This relatively lengthy data collection period reflects the time frame during which access to test results became increasingly available in the Netherlands.

We interviewed patients both with and without experience of real-time access to test results. In 2020 we were unable to find enough patients with experience, so we expanded our sample by interviewing more patients in 2021. We stopped interviewing when we reached data saturation, when we considered the sample size adequate. This is described more thoroughly (see above).

Results

The results section includes relevant quotes that effectively illustrate the themes and subthemes of the discourse. Suggestions from participants on how to mitigate stress, which is valuable for practical application of the findings

Thank you for your kind words.

Discussion

The discussion effectively summarizes the main findings and links them to the research question and provides specific recommendations for healthcare providers, policy makers, and technology designers. The findings are well contextualized within the existing body of research, highlighting both confirmations and new insights.

Some suggestions: The discussion is quite long and could potentially be condensed for improved readability.

Thank you for this suggestion. We added an extra heading, named “Practical implications” for improved readability. Furthermore, we have also taken the opportunity to improve the readability of the whole section. The full text of the condensed version is perhaps too long to be included here. But, to give an indication of the difference, the original text was 2128 words long; the revised text is 1819 words, a reduction of 309 words equal to one page (A4).

More discussion on how the findings might differ across demographic groups could be valuable.

Thank you for this comment. We have added this to the Limitations section:

> Third, we did not stratify our sample, which could account for the differences among discourses according to gender or type of result (sensitive/not sensitive, routine/diagnostic). Earlier studies on result types and real-time access have mixed findings [20,56]. For instance, in a study of 30 cancer patients accessing laboratory results in real time reduced anxiety [20]. Another study reported most patients preferred real-time access for less sensitive diagnoses (high cholesterol, strep throat, genetic disease, sexually transmitted disease) but preferred a time delay for sensitive results (Alzheimer’s disease, fetal miscarriage, cancer) [56]. Please note, however, that our microlevel discourse analysis aimed to describe discourses, not to explain them. We wanted to examine how individuals socially construct a new technological functionality, namely real-time access to test results via patient portals. Articulating the differences in the social constructs of different discourses, invites us to reflect upon the practical implications of each discourse. This in turn could inform the design and embedding process of patient portals. Further research could expand or enrich the three discourses by zooming in on the differences in demographics, such as type of test result, age and sensitivity of the result. For example, one scoping review showed that older patients, those unfamiliar with portals, and those with abnormal results or conditions like cancer, cardiovascular disease or depression use portals less often for radiology results and prefer direct communication with a physician [11]. These patients are likely to frame real-time access as a source of stress.

Consider discussing how patients could be involved in implementing the recommendations derived from this study.

Thank you for this suggestion. We have added this to the end of the Practical implications section:

> Healthcare organizations can involve patients in the implementation, integration and evaluation of policies related to real-time access to test results. The evaluation can take the form of action research [54], where patients and professionals co-create, evaluate and improve the processes around access to test results during the research process.

Thank you for your valuable comments and suggestions, which have done much to improve our paper.

Best regards, also on behalf of Anne Marie Weggelaar, Kees Ahaus, Bettine Pluut,

Pauline Hulter

Dear Dr. Bryan D. Steitz (reviewer 2),

Thank you very much for reviewing our paper. Here is our point-by-point response to your comments.

Thank you for the opportunity to review this manuscript. The manuscript reports a qualitative study of patient perspectives towards receiving test results via patient portal in The Netherlands. This study topic is timely and relevant to the journal readership. As written, there are several opportunities for improvement.
My primary concern is that the focus of the study, test results, does not provide enough context or nuance to fully understand the various patient attitudes and perceptions around the breadth of test results and clinical contexts. Prior research has shown that patient attitudes towards reviewing test results vary based on the type of result (e.g. lab test versus imaging), result sensitivity, prior discussion about the result and/or possible outcomes (e.g. anticipatory guidance), patient characteristics (e.g. attained education, existing health utilization), and numerous other factors. Without this context, it is presently difficult to contextualize the presented results with other, related, studies.

Thank you for your comment. We acknowledge that additional demographic variables could stratify our sample and we could indeed have also explored which discourses are dominant among specific participants. For example, the discourse *real-time access as source of stress* may be dominant in people with stressful checking behavior that may last until the test results are disclosed. This discourse would only emerge among participants with previous experience of portal use.

However, as the Introduction explains, the main purpose of a discourse analysis is not to explain differences in discourses on the basis of patient characteristics or other factors. Rather, the aim is to closely examine how individuals socially construct certain phenomena differently. In our study, we looked at the construction of new technological functionalities, namely real-time access to test results via patient portals. Highlighting differences in the social constructions of real-time access, i.e., articulating different discourses, invites us to reflect upon the practical implications of each discourse. This in turn could inform the design of patient portals and the processes in which these should be embedded. Consequently, we tried to design a sample as broad and balanced as possible [31] by including diverse participants with and without experience of real-time access to test results. In the end, each discourse we identified in our study articulates a different set of arguments and practices that appear to dominate when people engage with real-time access to test results [29]. As Deetz argues, the results of a discourse analysis can be evaluated by looking at how the discourse invites us to reflect on important differences [33,35]. We reflect on these differences and their implications in the Discussion. The above-mentioned premises of discourse analysis imply that demographic variables are not necessarily a central topic of interest. Of course, other kinds of future research, such

as quantitative studies, could focus more on the relation between patient characteristics and opinions of patient portals. Further research examining the three discourses in relation to the types of test results, sensitivity of the results, or patient characteristics could enrich our understanding of these discourses and their implications.

References

29. *Pluut, B. Differences that matter: developing critical insights into discourses of patient-centeredness. Medicine, Health Care and Philosophy 2016;19:501–515. Doi: 10.1007/s11019-016-9712-7.*

31. *Wetherell M, Taylor S, and Yates SJ. Discourse as Data. A guide for analysis. The Open University: Walton Hall. 2010. ISBN: 0-7619-7158-0*

33. *Deetz, S. Describing differences in approaches to organization science: Rethinking Burrell and Morgan and their legacy. Organization Science 1996;7(2): 191-207.*

35. *Phillips N, Hardy C. Managing multiple identities: discourse, legitimacy and resources in the UK refugee system. Organization; 1997;4(2):159-185. Doi: 10.1177/135050849742002.*

However, we have addressed your concern in the Limitations section:

> Third, we did not stratify our sample, which could account for the differences among discourses according to gender or type of result (sensitive/not sensitive, routine/diagnostic). Earlier studies on result types and real-time access have mixed findings [20,56]. For instance, in a study of 30 cancer patients accessing laboratory results in real time reduced anxiety [20]. Another study reported most patients preferred real-time access for less sensitive diagnoses (high cholesterol, strep throat, genetic disease, sexually transmitted disease) but preferred a time delay for sensitive results (Alzheimer's disease, fetal miscarriage, cancer) [56]. Please note, however, that our microlevel discourse analysis aimed to describe discourses, not to explain them. We wanted to examine how individuals socially construct a new technological functionality, namely real-time access to test results via patient portals. Articulating the differences in the social constructs of different discourses, invites us to reflect upon the practical implications of each discourse. This in turn could inform the design and embedding process of patient portals. Further research could expand or enrich the three discourses by zooming in on the differences in demographics, such as type of test result, age and sensitivity of the result. For example, one scoping review showed that older patients, those unfamiliar with portals, and those with abnormal results or conditions like cancer, cardiovascular disease or depression use portals less often for radiology results and prefer direct communication with a physician [11]. These patients are likely to frame real-time access as a source of stress.

Additionally:

In the Abstract, the Participants section suggests that only patients without experience to result review were interviewed, but the Setting section and manuscript conflict this. Please clarify whether patients who received test results were included in the study.

The Participants line in the Abstract states:

> Patients with experience (n=15) and without experience (n=13) of real-time access to test results on a patient portal.

In the second paragraph of the Introduction, the authors describe that providers can choose the time horizon for which test results are released. Please clarify that requirements for result release vary by country, and that the stated release strategy is specific to The Netherlands.
Thank you for your suggestion. We have revised the second paragraph as follows:

> National digital health patient portals vary by country, as do the requirements for disclosing test results [10]. In the Netherlands, healthcare providers can decide when patients can access test results on the portal. A recent scoping review shows that this is still an important topic [11].

Throughout the manuscript, the authors refer to "(dis)advantages" around accessing test results. It is unclear whether this refers to advantages and disadvantages, or only disadvantages. Please clarify throughout the text.

> Thank you for your comment. We meant advantages and disadvantages referring to "(dis)advantages". We addressed this throughout the whole manuscript by revising "(dis)advantages" to "advantages and disadvantages".

Table 2 presents participant characteristics including sex, age, and experience with portal. However, given the numerous patient characteristics that may influence perceptions about result review, it would be beneficial to expand this table to further describe the study population. Other possible metrics may include attained education, health utilization, and existing technology use.

Thanks for your comment. The Limitations section makes clear that we did not stratify our sample:

> Third, we did not stratify our sample, which could account for the differences among discourses according to gender or type of result (sensitive/not sensitive, routine/diagnostic). Earlier studies on result types and real-time access have mixed findings [20,56]. For instance, in a study of 30 cancer patients accessing laboratory results in real time reduced anxiety [20]. Another study reported most patients preferred real-time access for less sensitive diagnoses (high cholesterol, strep throat, genetic disease, sexually transmitted disease) but preferred a

> time delay for sensitive results (Alzheimer's disease, fetal miscarriage, cancer) [56]. Please note, however, that our microlevel discourse analysis aimed to describe discourses, not to explain them. We wanted to examine how individuals socially construct a new technological functionality, namely real-time access to test results via patient portals. Articulating the differences in the social constructs of different discourses, invites us to reflect upon the practical implications of each discourse. This in turn could inform the design and embedding process of patient portals.

Page 10, line 5: The inclusion criteria that patients "had or had not used a patient portal.." reads as irrelevant given that this criteria encompasses both possible options. Consider removing for clarity.

Thank you for your suggestion. We now fully explain our purposive sampling in the Method section:

> We used purposive and snowball sampling to find participants. We aimed to select patients with and without experience of real-time access to test results. Three researchers (PH, MB, BP) began by approaching their own networks [40], calling potential participants to explain the study and inviting them to an interview. At the end of interviews, we asked participants if they knew other patients we could interview (snowball method). In total, we included 28 participants (see Table 1 for participant characteristics).
>
> [Table 1]
>
> Aiming for a broadly inclusive participant sample [31] we included participants both with and without (never accessed) experience of real-time access to test results online. We felt it important to include diverse patients to understand the thoughts, expectations, needs, and wishes of patients without experience. We began interviewing participants without experience in March 2018. At the time, few Dutch healthcare organizations offered real-time access to test results [21]. We stopped interviewing when we reached data saturation (i.e., new data no longer provided new insights) at which point we felt the sample of patients without experience was adequate [41]. Two years later, more healthcare organizations offered real-time access, which in April 2020 enabled us to begin interviewing participants with experience. Again, when we reached data saturation in June 2021, we stopped interviewing. In total, we interviewed 13 participants without real-time access experience (8 females, 5 males) and 15 participants with real-time access experience (10 females, 5 males).

On page 13, line 27: This first sentence is redundant and should be revised for clarity.

Under Discourse 1 we revised the opening sentence to:

> *Real-time access as a source of stress* frames real-time access as a potential stressor caused by various reasons.

Throughout the Results, the authors refer generally to the number of participants who responded according to a particular theme. For example, on page 13, line 44 mentions "…participants were worried about interpreting…". Please consider quantifying the number of patients who responded according to the stated themes.

Thank you for your suggestion. Upon consideration, we think that quantifying the number of patients responding to each theme will not improve the quality of data presentation in our paper. Our study aim was to gain a deep understanding of patients' decision-making processes by unraveling three discourses on real-time access to test results, each of which articulates a different set of values, assumptions and arguments. Quantifying participants can lead to unwanted generalizations about how representative certain statements are of the entire group. In discourse analysis, it is often more important to focus on context and the variety of perspectives rather than on numerical data.

On page 30, line 46: Reference 41 appears to refer to a study about patient perceptions of receiving immediately released test results. This study does not present empiric data about patient notification preference as cited in the manuscript text. I suggest the following reference instead: "Impact of notification policy on patient-before-clinician review of immediately released test results"; https://doi.org/10.1093/jamia/ocad126.

Thank you for alerting us to this interesting paper. We have included it as a reference and have revised the sentence as follows:

> One study suggests two notification policies: immediate notifications for all results and only for patients who have opted in for notifications [44].

Since this study was conducted before, during, and after the COVID-19 pandemic, the authors should include the possibility that patient perspectives about receiving test results via patient portal may have changed in response to new or temporary practice patterns caused by the pandemic.

Thank you for this suggestion. We added this to the Limitations section:

> Fourth, we conducted this study before and during the Covid-19 pandemic. Patient perspectives on receiving test results via the patient portal may have shifted in response to the new and/or temporary online practices that emerged during the pandemic.

VERSION 2 - REVIEW

Reviewer 2

Name Steitz, Bryan D

Affiliation Department of Biomedical Informatics, Vanderbilt University Medical Center, Nashville, Tennessee.

Date 30-Oct-2024

COI

The authors have responded well to my prior comments. I have no additional suggestions. I look forward to seeing this manuscript in press.
